# Impact of time to revision total knee arthroplasty on outcomes following aseptic failure

**DOI:** 10.1186/s43019-023-00191-5

**Published:** 2023-05-30

**Authors:** Mackenzie A. Roof, Shankar Narayanan, Nathan Lorentz, Vinay K. Aggarwal, Morteza Meftah, Ran Schwarzkopf

**Affiliations:** Department of Orthopedic Surgery, NYU Langone Health, 301 East 17th Street, 15th Fl Suite 1518, New York, NY 10003 USA

**Keywords:** Revision total knee arthroplasty, Timing, Indications, Outcomes

## Abstract

**Introduction:**

Prior studies have demonstrated an association between time to revision total knee arthroplasty (rTKA) and indication; however, the impact of early versus late revision on post-operative outcomes has not been reported.

**Materials and methods:**

A retrospective, observational study examined patients who underwent unilateral, aseptic rTKA at an academic orthopedic hospital between 6/2011 and 4/2020 with > 1-year of follow-up. Patients were *early* revisions if they were revised within 2 years of primary TKA (pTKA) or *late* revisions if revised after greater than 2 years. Patient demographics, surgical factors, and post-operative outcomes were compared.

**Results:**

470 rTKA were included (199 early, 271 late). Early rTKA patients were younger by 2.5 years (*p* = 0.002). The predominant indications for *early* rTKA were instability (28.6%) and arthrofibrosis/stiffness (26.6%), and the predominant indications for *late* rTKA were aseptic loosening (45.8%) and instability (26.2%; *p* < 0.001). Late rTKA had longer operative times (119.20 ± 51.94 vs. 103.93 ± 44.66 min; *p* < 0.001). There were no differences in rTKA type, disposition, hospital length of stay, all-cause 90-day emergency department visits and readmissions, reoperations, and number of re-revisions.

**Conclusions:**

Aseptic rTKA performed before 2 years had different indications but demonstrated similar outcomes to those performed later. Early revisions had shorter surgical times, which could be attributed to differences in rTKA indication.

**Level of evidence:**

III, retrospective observational analysis.

**Supplementary Information:**

The online version contains supplementary material available at 10.1186/s43019-023-00191-5.

## Introduction

As the number of primary and revision TKA cases in the United States continue to rise, there is an increasing burden placed on both patients and the healthcare system to address the issue of TKA failure [1–3]. Early aseptic rTKA is of particular concern, as the cause of these failures can be multifactorial and difficult to address [4–6]. Loeffler et al. theorized that optimizing patients preoperatively and improving patient selection for primary arthroplasty may help address this growing problem [7].

Many indications for rTKA have been reported, with the most common etiologies including polyethylene wear, aseptic loosening, instability, and infection [8–12]. These causes have changed over time, with osteolysis and infection historically being the leading causes of TKA failure [9, 10, 12]. In a 2002 retrospective review, Sharkey et al. originally reported the predominant mechanism of TKA failure was polyethylene liner wear, accounting for 25% of all revisions [11]. However, in a 10-year update out of the same institution, other etiologies such as aseptic loosening (39.9%), infection (27.4%), instability (7.5%), and periprosthetic fractures (4.7%) had all surpassed polyethylene wear (3.5%) [13].

Indication for rTKA is tightly intertwined with time to rTKA. In Postler, et al.’s review of 402 rTKA, the most common indications for early index rTKA (< 2 years after pTKA) were infection, aseptic loosening, extensor mechanism issues, and arthrofibrosis, whereas infection, aseptic loosening, and periprosthetic fracture were the most common indications for late index rTKA (> 2 years after pTKA) [8]. Pietzrak, et al. excluded infection, periprosthetic fractures, and skin-related complications from their series, and found that early index rTKA (< 2 years after pTKA) were most commonly done for arthrofibrosis and patellar complications, whereas late index rTKA (> 2 years after pTKA) were most commonly done for aseptic loosening and tibiofemoral laxity [14].

Elucidating the effect of time to rTKA is important, as early rTKA are more likely to represent issues with preoperative planning prior to pTKA, as well as surgical decision making and acute postoperative patient management. Beyond the pTKA, early rTKA indications may require more expeditious rTKA, including infection, extensor mechanism disruption insufficiency and arthrofibrosis/stiffness. Pietzrak et al. examined 255 rTKA. These indications may require more expeditious rTKA, affecting the opportunity to appropriately optimize the patient prior to rTKA.

While previous studies have reported on the relationship between timing of rTKA and indication for the procedure, there is a paucity of literature describing the effect of time to rTKA on outcomes for aseptic failures. We therefore set out to investigate the effect of time from primary TKA on both indications and outcomes of aseptic TKA revisions. Given that earlier revisions are likely related to preoperative planning and intraoperative surgical decision making, we hypothesize that early revisions, performed within 2 years of primary replacement, will have inferior outcomes following rTKA compared to late rTKA, performed greater than 2 years following primary replacement. The cutoff of 2 years was utilized as multiple previously cited studies in the literature have used this timepoint to indicate early versus late rTKA [8, 14].

## Materials and methods

After receiving approval from he institutional review board (IRB), the rTKA database at a single, large, academic institution was retrospectively queried for all patients who underwent index, aseptic, unilateral rTKA between January 2011 and April 2020. Patients who underwent bilateral rTKA, rTKA for periprosthetic infection, or conversion arthroplasty from unicompartmental knee arthroplasty or previous fracture repair were excluded. Given the interest in outcomes following rTKA, only patients with at least 1 year of documented follow-up were included in the study.

Demographic information (age, sex, race, body mass index [BMI], American Society of Anesthesiology [ASA] score, and smoking status), admission data (implants revised, indication for revision, date of surgery, length of stay [LOS]), and surgical history (date of pTKA and index rTKA) were collected from our electronic medical record warehouse, Epic (Verona, WI). The indication for revision was documented by the operating surgeon at the time of the surgery. If multiple causes of failure were documented by the operating surgeon, then the charts were manually reviewed by one of the senior authors to determine the primary indication for revision. Implants revised included femoral component, polyethylene liner, tibial baseplate, and patellar button. Full rTKA required revision of the femoral component, polyethylene liner, tibial baseplate. Femoral rTKA required revision of the femoral component without revision of the tibial baseplate. Tibial rTKA required revision of the tibial baseplate without revision of the femoral component. Polyethylene liner exchanges required revision of the polyethylene liner without revision of the femoral component or tibial baseplate. Patellar rTKA required revision of the patellar component without revision of the femoral, polyethylene liner, or tibial components. Hospital LOS was determined as the whole number of days between admission and discharge. Surgical time was calculated as the time between incision start and incision close. Discharge disposition categories included discharge to home with either self-care or home health services, discharge to an acute rehabilitation facility, and discharge to a skilled nursing facility. Emergency department (ED) and readmissions within 90-days, all reoperations, and all re-revisions were dichotomized to “yes” or “no”. A reoperation was defined as any procedure requiring return to the operating room following the rTKA that was related to the ipsilateral knee and did not require a change in implants. A re-revision was defined as any procedure requiring return to the operating room following the rTKA that was related to the ipsilateral knee and did require a change in implants.

For patients who had the precise date of their pTKA in the electronic medical record, time to rTKA was calculated as the whole number of days between the pTKA and the index rTKA. For patients who only had the year of pTKA documented in the chart, time to rTKA was calculated as the whole number of years between the pTKA and the index rTKA. For patients who had the month and the year of pTKA documented in the chart, this was converted to a date format of the first of the month (ex: October 2016 was converted to October 1, 2016) and time to rTKA was calculated as the whole number of days between the pTKA and the index rTKA. Patients were dichotomized to early rTKA if they underwent index rTKA within 2 years/730 days of pTKA. Patients were dichotomized to late rTKA if they underwent rTKA after 2 years/730 days following pTKA.

### Statistical analysis

Categorical variables were analyzed using a chi-square analysis and continuous variables were analyzed using an Independent Samples *T*-Test. IBM SPSS Statistics Version 25 (Armonk, NY) was utilized for the statistical analyses. The alpha level was set at *p* < 0.05 for statistical significance.

## Results

### Patient selection

The rTKA database at our institution was reviewed for rTKA cases performed between June 2011 and April 2020. One thousand, six-hundred, and seventy-one rTKA were identified. Five hundred and twenty-seven rTKA (31.5%) were excluded for revision for septic reasons. Six rTKA (0.36%) were excluded for simultaneous bilateral revisions. Sixty-nine rTKA (4.1%) were excluded for conversion arthroplasty or bicompartmental or unicompartmental knee arthroplasty revision. Eight rTKA (0.48%) were excluded for patella component removal only. 177 were excluded for being a re-revision surgery (10.6%). Three-hundred and ninety-eight rTKA (23.8%) were excluded for inadequate follow-up. Sixteen rTKA (0.96%) were excluded for lacking the date of pTKA in the medical chart.

### Demographics

In total, 470 aseptic, unilateral, index rTKA in 463 patients were identified. Of these, 199 rTKA (42.3%) were early and 271 (57.7%) were late. Patients who underwent late rTKA were significantly older (64.6 ± 8.6 years) as compared to patients who underwent early rTKA (62.0 ± 9.08 years, *p* = 0.002). There were no statistically significant differences in sex, BMI, ASA score, smoking status, and laterality between the two cohorts (Table 1). For early rTKA, mean time to revision was 1.0 ± 0.5 years. For late rTKA, mean time to revision was 7.8 ± 5.7 years.

**Table 1:** Demographic information

Variable	Early revisions (*n* = 199)	Late revisions (*n* = 271)	*P*-value
Age [mean years (SD)]^c^	62.04 (9.08)	64.59 (8.56)	0.002**
Sex [*n* (%)]^a^	–	–	0.687
Male	63 (31.7)	81 (29.9)	
Female	136 (68.3)	190 (70.1)	
Race [*n* (%)]^b^	–	–	0.077
White	110 (55.6)	132 (49.1)	
Black	45 (22.7)	87 (32.3)	
Asian	2 (1.0)	6 (2.2)	
Other	41 (20.7)	44 (16.4)	
BMI [mean kg/m^2^ (SD)]^c^	32.47 (6.53)	33.60 (6.95)	0.084
ASA score [*n* (%)]^b^	–	–	0.057
1	0 (0.0)	6 (2.4)	
2	102 (56.0)	131 (51.6)	
3	79 (43.4)	110 (43.3)	
4	1 (0.5)	7 (2.8)	
Smoking status [*n* (%)]^b^	–	–	0.338
Never	120 (60.3)	147 (54.2)	
Former	64 (32.2)	105 (38.7)	
Current	15 (7.5)	19 (7.0)	
Laterality [*n* (%)]^a^			0.851
Right	108 (54.3)	150 (55.4)	
Left	91 (45.7)	121 (44.6)	

Categorical variables analyzed by Fisher’s exact test (^a^) or Chi square test (^b^), where appropriate; continuous variables analyzed by Independent Samples *T*-Test (^c^)

**p < 0.01

### Surgical information

When examining all aseptic rTKA types together (full, femoral, tibial, polyethylene liner, and patellar), there were no significant differences between the time cohorts with respect to rTKA type (*p* = 0.071). The most common indications for early revisions were instability/dislocation (28.6%), arthrofibrosis/stiffness (26.6%), and aseptic loosening (26.1%), whereas the most common indications for late rTKA were aseptic loosening (45.8%), instability (26.2%), and arthrofibrosis/stiffness (9.6%; *p* < 0.001; Table 2).

**Table 2:** Surgical information—all revisions

Variable	Early revisions (*n* = 199)	Late revisions (*n* = 271)	*P*-value
Type of revision [n (%)]^a^	–	–	0.071
Full	95 (47.7)	158 (58.3)	
Femoral	21 (10.6)	13 (4.8)	
Tibial	24 (12.1)	29 (10.7)	
Liner	48 (24.1)	55 (20.3)	
Patellar	11 (5.5)	15 (5.9)	
Reason for revision [n (%)]^a^	–	–	< 0.001*
Arthrofibrosis/stiffness/ankylosis	53 (26.6)	26 (9.6)	
Aseptic loosening	52 (26.1)	124 (45.8)	
Component malpositioning	8 (4.0)	6 (2.2)	
Extensor mechanism/patellar clunk	11 (5.5)	4 (1.5)	
Periprosthetic fracture	8 (4.0)	7 (2.6)	
Implant failure	4 (2.0)	10 (3.7)	
Instability/dislocation	57 (28.6)	71 (26.2)	
Liner wear	1 (0.5	17 (6.3)	
Nickel metal allergy	5 (2.5)	3 (1.1)	
Patellofemoral osteoarthritis	5 (2.5)	3 (1.1)	
Osteolysis	0 (0.0)	2 (0.7)	
Surgical time [mean minutes (SD)]^b^	103.93 (44.66)	119.20 (51.94)	0.001*

Categorical variables analyzed by Chi square test (^a^), where appropriate; continuous variables analyzed by Independent Samples *T*-Test (^b^)

*p < 0.01

### Postoperative outcomes

Late rTKA had significantly longer operative times by 16 min (119.2 ± 51.9 min vs. 103.9 ± 44.7 min; *p* = 0.001). There were no differences between the two cohorts with respect to discharge disposition, hospital LOS, all-cause 90-day ED visits and readmissions, reoperations, re-revisions, reoperations/re-revisions for infection, and number of re-revisions (Table 3). For early rTKA, mean follow-up was 3.1 ± 1.8 years. For late rTKA, mean follow-up was 2.9 ± 1.8 years. A breakdown of the outcome results by rTKA type excluding the patients with less than 1 year of follow-up can be found in Additional file 1: Appendices 1–10.

**Table 3:** Outcome information—all revisions

Variable	Early revisions (*n* = 199)	Late revisions (*n* = 271)	*P*-value
Length of stay [mean days (SD)]	6.38 (17.04)	8.14 (19.68)	0.301
Discharge disposition [*n* (%)]^b^	–	–	0.347
Home	157 (78.9)	211 (77.9)	
Acute rehabilitation facility	11 (5.5)	10 (3.7)	
Skill*e*d Nursing Facility	31 (15.6)	47 (17.3)	
Other	0 (0.0)	3 (1.1)	
All cause 90-day ED visit [*n* (%)]^a^	9 (4.5)	11 (4.1)	0.821
All cause 90-day readmission [*n* (%)]^a^	16 (8.0)	30 (11.1)	0.346
Reoperation [*n* (%)]^a^	22 (11.1)	20 (7.4)	0.191
Re-revision [*n* (%)]^a^	43 (21.6)	44 (16.2)	0.150
Number of re-revisions [mean re-revisions (SD)]	0.32 (0.74)	0.24 (0.62)	0.207
Mortality [*n* (%)]^a^	0 (0.0)	2 (0.7)	0.511

Categorical variables analyzed by Chi square test (^a^), where appropriate; continuous variables analyzed by Independent Samples* T*-Test (^b^)

*p < 0.01

## Discussion

The present study demonstrated that early rTKA patients were younger by about 2.5 years and were more likely to undergo rTKA for instability/dislocation (28.6%), arthrofibrosis/stiffness (26.6%), and aseptic loosening (26.1%) as compared to late rTKA patients, who were more often revised for aseptic loosening (45.8%), instability (26.2%), and arthrofibrosis/stiffness (9.6%). Despite differences in indication, there were no differences in rTKA type or short-term outcomes following rTKA.

The finding that early rTKA patients were significantly younger than their late rTKA counterparts is consistent with findings in the literature. Walker-Santiago, et al. conducted a retrospective review comparing 147 patients aged 55 years or less to 276 patients aged 60–75 years and found that the younger cohort had a significantly higher rate of early rTKA within 2 years (*p* < 0.001) [15]. Other reports have demonstrated similar findings [16, 17]. The reason for this finding likely multifactorial. However, younger patient are typically more active than older patients, which may lead to higher demand placed on their prosthetic joint, leading to earlier failure.

In our series, the predominant indications for all revisions, regardless of timing, included aseptic loosening and instability. This is in line with reports in the literature with rates of aseptic loosening and instability at 29.8% and 6.2% respectively, in worldwide registries [8]. However, when categorizing revisions by timing, early rTKA had a greater proportion indicated for instability, whereas late rTKA had a greater proportion indicated for aseptic loosening. This is consistent with the literature which demonstrates instability to be an early mode of TKA failure [18, 19]. Loosening can be an early complication of TKA due to failure of the implant to gain appropriate fixation or a late complication reflecting loss of fixation due to bone resorption [20]. Our study supported this claim, as aseptic loosening was a common indication of both early and late rTKA. However, the late rTKA cohort (45.8%) had nearly double the proportion of cases revised for aseptic loosening as compared to the early rTKA cohort (26.1%), supporting the hypotheses that loosening results from mechanical loss of fixation or biological loss of fixation due to either particle-induced osteolysis or mechanical loosening/debonding, both of which typically develop gradually over time [20, 21].

When examining surgical characteristics, we found that the late rTKA cohort had significantly longer operative times than the early rTKA cohort by about 16 min. This could in part be due to differences in surgical indications between early and late rTKA. Additionally, studies have demonstrated that longer operative times can result in increased post-operative complications in patients undergoing rTKA [22–24]. However, in the present cohort, no significant difference in other outcome variables were observed between the two cohorts in the present study (i.e. discharge disposition, hospital LOS, all-cause 90-day emergency department visits and readmissions, reoperations, re-revisions, reoperations/re-revisions for infection, and number of re-revisions). From these observations, we may reasonably conclude that early and late aseptic rTKA result in similar postoperative outcomes.

Although we did not assess cost implications in the current study, differences between early and late rTKA can also be viewed from a medico-economic perspective. A cost utility model demonstrated that early TKA cost substantially more than late TKA with a minimal gain in quality of life years [25]. While this should not affect a surgeons’ decision on when to schedule a patient for surgery, it is valuable in guiding the decision-making of healthcare institutions. Additionally, previous studies have shown that revisions for different indications have a wide range in cost to the healthcare system, ranging from $13,900–$29,200 for aseptic rTKA to $24,000–$38,100 for septic rTKA [26]. We were unable to identify any literature comparing costs for early versus late rTKA. Future lines of inquiry should focus on quantifying these costs to ascertain whether there is a difference in cost to the healthcare system depending on the timing of rTKA.

### Limitations

The retrospective observational design of the present studies has several inherent limitations, including the possibility for collection error and selection bias. This collection error includes imprecise recording of pTKA date. Fortunately, only 16 of 470 rTKA (3.4%) had solely the year of pTKA recorded that resulted in a time to rTKA of exactly 2 years. This small percentage of rTKA were categorized as early revisions. Secondly, given that this study was conducted at one large, academic, urban tertiary referral center that performs a high volume of rTKA, these results may not necessarily be generalizable to smaller community centers in other areas of the country. Thirdly, we do not have metrics on patient satisfaction scores, such as patient-reported outcome measures (PROM), so we are unable to comment on the effect of early vs. late rTKA on patient satisfaction scores. Lastly, our study was limited by the amount of individual patient follow-up. This limitation was somewhat mitigated through excluding all patients with less than 1 year of follow-up.

## Conclusion

The current study illustrated that instability was the predominant surgical indication for early aseptic revision, whereas aseptic loosening dominated the indications for late aseptic TKA revisions. Even with these variances, no significant difference was observed in short-term post-operative outcomes between early and late rTKA.

## Supplementary Information

**Additional file 1.** Additional data tables.

## Data Availability

All data and material is available for review if requested by the reviewers/editors.
