# Supplementary material for: Impact of time to revision total knee arthroplasty on outcomes following aseptic failure
Source: Knee Surg Relat Res. 2023 May 30;35:15. doi: 10.1186/s43019-023-00191-5 (PMC10230807; doi:10.1186/s43019-023-00191-5)
Supplement: Supplementary file 1 — Additional file 1. Additional data tables. [file 43019_2023_191_MOESM1_ESM.docx]

**Appendices:**

| Appendix 1. Surgical information – full revisions. | | | |
| --- | --- | --- | --- |
| **Variable** | **Early revisions (n=95)** | **Late revisions (n=158)** | **P-value** |
| *Reason for revision [n (%)]*• | -- | -- | <0.001** |
| Arthrofibrosis/stiffness/  ankylosis | 30 (31.6) | 13 (8.2) |  |
| Aseptic loosening | 19 (20.0) | 81 (51.3) |  |
| Component malpositioning | 5 (5.3) | 5 (3.2) |  |
| Extensor mechanism/patellar clunk | 4 (4.2) | 2 (1.3) |  |
| Periprosthetic fracture | 4 (4.2) | 4 (2.5) |  |
| Implant failure | 2 (2.1) | 4 (2.5) |  |
| Instability/dislocation | 28 (29.5) | 38 (24.1) |  |
| Liner wear | 1 (1.1) | 6 (3.8) |  |
| Nickel metal allergy | 0 (0.0) | 1 (0.6) |  |
| Osteoarthritis | 2 (2.1) | 2 (1.3) |  |
| Osteolysis | 0 (0.0) | 2 (1.3) |  |
| *Surgical time [mean minutes (SD)]*∂ | 130.02 (37.64) | 142.03 (46.70) | 0.035* |
| Categorical variables analyzed by Fisher’s exact test (◊) or Chi square test (•), where appropriate; continuous variables analyzed by Independent Samples T-Test (∂).  * p<0.05, ** p<0.01 | | | |

| Appendix 2. Outcome information – full revisions. | | | |
| --- | --- | --- | --- |
| **Variable** | **Early revisions (n=95)** | **Late revisions (n=158)** | **P-value** |
| *Length of stay*  *[mean days (SD)]* | 6.37 (14.00) | 10.58 (23.26) | 0.074 |
| *Discharge disposition*  *[n (%)]* | -- | -- | 0.478 |
| Home | 76 (80.0) | 117 (74.1) |  |
| Acute Rehabilitation Facility | 6 (6.3) | 8 (5.1) |  |
| Skilled Nursing Facility | 13 (13.7) | 32 (20.3) |  |
| Other | 0 (0.0) | 1 (0.6) |  |
| *All cause 90-day ED visit [n (%)]*◊ | 6 (6.3) | 7 (4.4) | 0.563 |
| *All cause 90-day Readmission [n (%)]*◊ | 9 (9.5) | 21 (13.3) | 0.426 |
| *Reoperation [n (%)]*◊ | 15 (15.8) | 15 (9.5) | 0.160 |
| *Re-revision [n (%)]*◊ | 16 (16.8) | 28 (17.7) | 1.000 |
| *Number of re-revisions [mean re-revisions (SD)]* | 0.27 (0.750) | 0.27 (0.64) | 0.930 |
| Categorical variables analyzed by Fisher’s exact test (◊) or Chi square test (•), where appropriate; continuous variables analyzed by Independent Samples T-Test (∂).  * p<0.05  ** p<0.01 | | | |

| Appendix 3. Surgical information – femoral revisions. | | | |
| --- | --- | --- | --- |
| **Variable** | **Early revisions (n=21)** | **Late revisions (n=13)** | **P-value** |
| *Reason for revision [n (%)]*• | -- | -- | 0.007** |
| Arthrofibrosis/stiffness/  Ankylosis | 11 (52.4) | 1 (7.7) |  |
| Aseptic loosening | 1 (4.8) | 5 (38.5) |  |
| Component malpositioning | 2 (9.5) | 0 (0.0) |  |
| Extensor mechanism/patellar clunk | 3 (14.3) | 0 (0.0) |  |
| Periprosthetic fracture | 3 (14.3) | 0 (0.0) |  |
| Implant failure | 0 (0.0) | 1 (7.7) |  |
| Instability/dislocation | 2 (9.5) | 5 (38.5) |  |
| *Surgical time [mean minutes (SD)]*∂ | 144.92 (49.21) | 92.62 (25.18) | 0.003** |
| Categorical variables analyzed by Fisher’s exact test (◊) or Chi square test (•), where appropriate; continuous variables analyzed by Independent Samples T-Test (∂).  * p<0.05  ** p<0.01 | | | |

| Appendix 4. Outcome information – femoral revisions. | | | |
| --- | --- | --- | --- |
| **Variable** | **Early revisions (n=21)** | **Late revisions (n=13)** | **P-value** |
| *Length of stay*  *[mean days (SD)]*∂ | 13.24 (34.47) | 3.23 (0.73) | 0.198 |
| *Discharge disposition*  *[n (%)]*• | -- | -- | 0.263 |
| Home | 15 (71.4) | 7 (53.8) |  |
| Acute Rehabilitation Facility | 3 (14.3) | 1 (7.7) |  |
| Skilled Nursing Facility | 3 (14.3) | 5 (38.5) |  |
| *All cause 90-day ED visit [n (%)]*◊ | 1 (4.8) | 0 (0.0) | 1.000 |
| *All cause 90-day Readmission [n (%)]*◊ | 1 (4.8) | 0 (0.0) | 1.000 |
| *Reoperation [n (%)]*◊ | 2 (9.5) | 0 (0.0) | 0.513 |
| *Re-revision [n (%)]*◊ | 4 (19.0) | 2 (15.4) | 1.000 |
| *Number of re-revisions [mean re-revisions (SD)]*∂ | 0.24 (0.54) | 0.15 (0.38) | 0.625 |
| Categorical variables analyzed by Fisher’s exact test (◊) or Chi square test (•), where appropriate; continuous variables analyzed by Independent Samples T-Test (∂).  * p<0.05  ** p<0.01 | | | |

| Appendix 5. Surgical information – tibial revisions. | | | |
| --- | --- | --- | --- |
| **Variable** | **Early revisions (n=24)** | **Late revisions (n=29)** | **P-value** |
| *Reason for revision [n (%)]*• | -- | -- | 0.719 |
| Arthrofibrosis/stiffness/  Ankylosis | 1 (4.2) | 2 (6.9) |  |
| Aseptic loosening | 20 (83.3) | 23 (79.3) |  |
| Component malpositioning | 1 (4.2) | 0 (0.0) |  |
| Periprosthetic fracture | 1 (4.2) | 1 (3.4) |  |
| Instability/dislocation | 1 (4.2) | 3 (10.3) |  |
| *Surgical time [mean minutes (SD)]*∂ | 108.45 (26.88) | 108.50 (35.18) | 0.995 |
| Categorical variables analyzed by Fisher’s exact test (◊) or Chi square test (•), where appropriate; continuous variables analyzed by Independent Samples T-Test (∂).  * p<0.05  ** p<0.01 | | | |

| Appendix 6. Outcome information – tibial revisions. | | | |
| --- | --- | --- | --- |
| **Variable** | **Early revisions (n=24)** | **Late revisions (n=29)** | **P-value** |
| *Length of stay*  *[mean days (SD)]*∂ | 2.79 (1.50) | 11.00 (23.71) | 0.073 |
| *Discharge disposition*  *[n (%)]*• | -- | -- | 0.414 |
| Home | 19 (79.2) | 22 (75.9) |  |
| Skilled Nursing Facility | 5 (20.8) | 5 (17.2) |  |
| Other | 0 (0.0) | 2 (6.9) |  |
| *All cause 90-day ED visit [n (%)]*◊ | 0 (0.0) | 0 (0.0) | N/A |
| *All cause 90-day Readmission [n (%)]*◊ | 2 (8.3) | 1 (3.4) | 0.584 |
| *Reoperation [n (%)]*◊ | 0 (0.0) | 0 (0.0) | N/A |
| *Re-revision [n (%)]*◊ | 3 (12.5) | 2 (6.9) | 0.649 |
| *Number of re-revisions [mean re-revisions (SD)]*∂ | 0.17 (0.48) | 0.14 (0.58) | 0.847 |
| Categorical variables analyzed by Fisher’s exact test (◊) or Chi square test (•), where appropriate; continuous variables analyzed by Independent Samples T-Test (∂).  * p<0.05  ** p<0.01 | | | |

| Appendix 7. Surgical information – liner revisions. | | | |
| --- | --- | --- | --- |
| **Variable** | **Early revisions (n=48)** | **Late revisions (n=55)** | **P-value** |
| *Reason for revision [n (%)]*• | -- | -- | 0.028* |
| Arthrofibrosis/stiffness/  Ankylosis | 11 (22.9) | 10 (18.2) |  |
| Aseptic loosening | 5 (10.4) | 4 (7.3) |  |
| Extensor mechanism/patellar clunk | 3 (6.3) | 1 (1.8) |  |
| Periprosthetic fracture | 1 (2.1) | 0 (0.0) |  |
| Implant failure | 2 (4.2) | 5 (9.1) |  |
| Instability/dislocation | 26 (54.2) | 24 (43.6) |  |
| Liner wear | 0 (0.0) | 11 (20.0) |  |
| *Surgical time [mean minutes (SD)]*∂ | 67.13 (33.54) | 66.07 (25.99) | 0.861 |
| Categorical variables analyzed by Fisher’s exact test (◊) or Chi square test (•), where appropriate; continuous variables analyzed by Independent Samples T-Test (∂).  * p<0.05  ** p<0.01 | | | |

| Appendix 8. Outcome information – liner revisions. | | | |
| --- | --- | --- | --- |
| **Variable** | **Early revisions (n=48)** | **Late revisions (n=55)** | **P-value** |
| *Length of stay*  *[mean days (SD)]* | 5.98 (17.15) | 2.45 (1.09) | 0.162 |
| *Discharge disposition*  *[n (%)]*• | -- | -- | 0.176 |
| Home | 39 (81.3) | 51 (92.7) |  |
| Acute Rehabilitation Facility | 1 (2.1) | 0 (0.0) |  |
| Skilled Nursing Facility | 8 (16.7) | 4 (7.3) |  |
| *All cause 90-day ED visit [n (%)]*◊ | 2 (4.2) | 3 (5.5) | 1.000 |
| *All cause 90-day Readmission [n (%)]*◊ | 3 (6.3) | 7 (12.7) | 0.331 |
| *Reoperation [n (%)]*◊ | 3 (6.3) | 3 (5.5) | 1.000 |
| *Re-revision [n (%)]*◊ | 15 (31.3) | 11 (20.0) | 0.256 |
| *Number of re-revisions [mean re-revisions (SD)]* | 0.38 (0.64) | 0.29 (0.69) | 0.523 |
| Categorical variables analyzed by Fisher’s exact test (◊) or Chi square test (•), where appropriate; continuous variables analyzed by Independent Samples T-Test (∂).  * p<0.05  ** p<0.01 | | | |

| Appendix 9. Surgical information – patellar revisions. | | | |
| --- | --- | --- | --- |
| **Variable** | **Early revisions (n=11)** | **Late revisions (n=16)** | **P-value** |
| *Reason for revision [n (%)]*• |  |  | 0.535 |
| Aseptic loosening | 7 (63.6) | 11 (68.8) |  |
| Component malpositioning | 0 (0.0) | 1 (6.3) |  |
| Extensor mechanism/patellar clunk | 1 (9.1) | 1 (6.3) |  |
| Periprosthetic fracture | 0 (0.0) | 1 (6.3) |  |
| Instability/dislocation | 0 (0.0) | 1 (6.3) |  |
| Osteoarthritis | 3 (27.3) | 1 (6.3) |  |
| *Surgical time [mean minutes (SD)]*∂ | 50.91 (19.85) | 74.94 (35.83) | 0.055 |
| Categorical variables analyzed by Fisher’s exact test (◊) or Chi square test (•), where appropriate; continuous variables analyzed by Independent Samples T-Test (∂).  * p<0.05  ** p<0.01 | | | |

| Appendix 10. Outcome information – patellar revisions. | | | |
| --- | --- | --- | --- |
| **Variable** | **Early revisions (n=11)** | **Late revisions (n=16)** | **P-value** |
| *Length of stay*  *[mean days (SD)]* | 2.91 (0.70) | 2.38 (1.5) | 0.283 |
| *Discharge disposition*  *[n (%)]*• | -- | -- | 0.583 |
| Home | 8 (72.7) | 14 (87.5) |  |
| Acute Rehabilitation Facility | 1 (9.1) | 1 (6.3) |  |
| Skilled Nursing Facility | 2 (18.2) | 1 (6.3) |  |
| *All cause 90-day ED visit [n (%)]*◊ | 0 (0.0) | 1 (6.3) | 1.000 |
| *All cause 90-day Readmission [n (%)]*◊ | 1 (9.1) | 1 (6.3) | 1.000 |
| *Reoperation [n (%)]*◊ | 2 (18.2) | 2 (12.5) | 1.000 |
| *Re-revision [n (%)]*◊ | 5 (45.5) | 1 (6.3) | 0.027* |
| *Number of re-revisions [mean re-revisions (SD)]* | 1.00 (1.41) | 0.06 (0.25) | 0.054 |
| Categorical variables analyzed by Fisher’s exact test (◊) or Chi square test (•), where appropriate; continuous variables analyzed by Independent Samples T-Test (∂).  * p<0.05  ** p<0.01 | | | |
